# Three Cases of Diphtheria Treated with Ruffer's Curative Serum

**Published:** 1895-12-28

**Authors:** W. D. Haslam


### Medical Progress and Hospital Clinics.

*[The Editor will be glad to receive offers of co-operation and contributions from members of the profession. All letters should be addressed to* The Editor, at the Office, 428, Strand, London, W.C.]

### THREE CASES OF DIPHTHERIA TREATED WITH RUFFER'S CURATIVE SERUM.

By W. D. Haslam, M.D. (Brux.), M.R.C.S.

**Case I.**—Friday, September 27th, 1895: M. W., æt. 12; school girl; tall for her age, but delicate; was taken ill yesterday with shivering and headache. To-day I found the patient in bed, breathing heavily, with flushed face, altered voice, and prominent enlargement of the cervical glands; her breath was very offensive, and she complained of sore throat, stiff neck, pain on swallowing. Temperature was 103; pulse, 130; tongue coated. Both tonsils were inflamed, and covered with well-marked membrane. There was also a patch on the root of the uvula extending to the left on the soft palate. Respiration was oral, the nares were obstructed. ℞. Tinct. ferri. perchlor., ʒi.; potass chlor. gr. 80; glycerine, ʒiii.; aquæ ad. ℥viii. One teaspoonful every hour. ℞. Euchlorine, ℥i. To swab the throat every hour. Suitable diet, &c. Nine p.m.—Injected 10 c.c. of antitoxic serum. Patient seems in a serious condition.

September 28th: Last night the patient was restless and delirous; bowels acted twice. Temperature at eight a.m. 101·6; pulse, 118, small and weak. There is a profuse discharge from the nostrils. Respiration is oral and noisy. At noon repeated injection of 10 c.c. of the serum. During the day the patient was very drowsy and at times delirious. Was removed to the Sanitary Hospital.

September 29th: Eight a.m., passed a restless night with muttering delirium; tried to get out of bed repeatedly. Membrane breaking up and becoming detached. Temperature at eight a.m. 100; pulse, 108; respiration, 20. Discharge continues from nares and is thicker; administered a third injection of 10 c.c. of antitoxic serum. Eight p.m., during the day patient was quieter, less delirious, but very drowsy, and complained of being disturbed so often for administration of nutrition and medicine; she is taking brandy ʒss. every four hours with milk. There are no new patches. Respiration not so noisy. Urine contains a trace of albumen.

September 30th: Patient has passed a quieter night, sleeping soundly at intervals. Nurse says "she seems quite herself this morning." At eight a.m. temperature was normal; pulse, 104; respiration, 20; no albuminuria. At noon temperature rose to 100, but was normal again at nine p.m.; the throat is clearing, and the glandular enlargements much decreased in size.

October 1st: Passed a good night and slept well. Throat looks cleaner; temperature, 99·2; not much discharge from nose; patient feels hungry. At eight p.m. temperature normal; pulse 98; stronger.

October 3rd: Convalescent.

October 5th: A new train of symptoms presented themselves, which are attributable to the serum. On the face, trunk, and limbs, are patches of urticaria; temperature normal, but much itching.

October 8th: Urticaria subsiding, but leaving behind large erythematous patches, which do not itch; patient was much improved and feeling well, until in the afternoon, about three, she complained of pains in chest; temperature rose to 102·6. At eight p.m. temperature was normal, pain gone.

October 9th: Temperature was normal at eight a.m.; patient felt fairly well after a good night, and was anxious to get up. At 11 a.m., she complained of pain in both wrists; temperature, 99·4; pulse, 108; tongue coated. Painful wrists to be wrapped round with cotton wool and oil silk; ordered a saline mixture. Half-past nine, was sent for, and found both ankles puffed up and exceedingly painful; the œdema extended over the dorsum of each foot, and patient could not bear the weight of the bedclothes. The whole body—limbs, trunk, and face were more or less covered with a red erythematous rash, and the temperature had risen to 103; pulse, 108. Urine sp. gr. 1,010, contained albumen, one-sixth; skin dry, hot, and pungent. There had been no headache, shivering, or vomiting. Throat looks well, and the remnants of the enlarged glands can be detected. Ankle joints treated as the wrists.

October 10th: Restless night, but little sleep. Temperature at eight a.m. 102·2; pulse, 112; tongue coated. Urine sp. gr. 1,020; plentiful albumen. Eruption inclined to fade. To have a purge and a turpentine stupe to the lumbar regions.

October 11th: Patient slept at intervals; muttering at times. Eight a.m., temperature, 102; pulse, 112, Improved during the day. Eight p.m., temperature, 101·2; pulse, 92.

October 13th: Temperature, pulse, and urine normal.

October 15th: Patient got up for the first time. Was discharged October 26th. No sequelæ.

N.B.—A specimen of the membrane from this case was examined at the British Institute of Preventive Medicine, and the bacillus diphtheriæ was isolated.

**Case II.**—October 1st: E. W., æt. 18, cook; lives at same house as No. 1 case; a strong, florid girl. Was taken ill yesterday with sore throat. Is very nervous about herself. At half-past two to-day there is slight redness of the soft palate and tonsils, but no sign of membrane. The glands are not enlarged, and the temperature is normal. There is, however, offensive breath, and a pulse of 130. Ordered a mixture of iron and chlorate of potash.

October 2nd (half-past eleven a.m.): Has shivered at times since yesterday; no headache. Temperature, 100; pulse, 120. Small patch on left tonsil. Was removed to the Sanitary Hospital. Half-past eight: Temperature, 100·5; pulse, 120. White patch on left tonsil distinct and diagnostic. No enlarged salivary glands. Complains much of sore throat. Injected 12 cc. of Ruffer's serum; is to continue with the iron mixture.

October 3rd: Restless night; throat painful, membrane thicker and spreading over left tonsil. Eight a.m.: Temperature, 102·4; pulse, 112; respiration, 16. Fresh patch on pharynx behind uvula. Complains of headache, giddiness, and sore throat. Eight p.m.; Temperature, 102·6; pulse, 116. Membrane disintegrating, no extension of membrane. Feels better. Administered a second injection of serum 12 cc.

October 4th: Patient has had a better night; sleeping at intervals. Eight a.m.: Temperature, 100·2; pulse 96. Is menstruating normally; throat clearing; general condition is improved.

October 5th: Passed a comfortable night; feels better. Temperature normal; pulse, 84.

October 6th: Patient passed a large round worm in her motion. Temperature normal; pulse, 84.

October 7th: Convalescent.

October 9th: Has pain in back and shoulders; feels giddy. Eight a.m.: temperature risen to 102·4; pulse, 116.

October 10th: Pains better. Temperature went down to normal by evening.

October 12th: Well again.

October 19th: Went home. No sequelæ.

**Case III.**—October 7th: E. B., æt. 16. Was taken ill yesterday with sore throat and stiff neck. Attack commenced with shivering. Patient's face is flushed, breath offensive. Temperature, 101·6; pulse, 144; tongue coated. Both tonsils are inflamed and covered with thin white patches of membrane. There is extreme enlargement of the left tonsil, which is partly chronic. At half-past eight temperature 102; pulse, 118. No enlargement of salivary glands. Injected 10 cc. Ruffer's serum.

October 8th: Slept well last night. Temperature, 101; pulse, 116. Complains of sore throat and pain on swallowing her food. Half-past eight: Temperature, 102·4; pulse, 120.

October 9th: Feels much better this morning. Half-past eight: Temperature, 99·4; pulse, 112; membrane disintegrating. Has headache. Half-past eight: Temperature, 100; pulse, 92. To have calomel gr. iij.

October 10th: Headache lasted all night, keeping the patient awake and restless. Towards the morning the calomel acted and headache disappeared soon after. At eight a.m. temperature was 98·6; pulse, 112. Throat clean.

October 11th: Temperature and pulse normal; convalescent.

October 12th: Left tonsil excised under cocaine.

October 25th: Patient went home, cured.

**Remarks.**—There are a few points accruing to these cases which I should like briefly to touch upon. All these cases were started in the same house, the diagnosis was confirmed by the microscope in Nos. 1 and 2, and they were each removed to the Sanitary Hospital without delay. There was no enlargement whatever of the salivary glands in Nos. 2 and 3, but this was most marked in No. 1. The breath was offensive in all three when first seen. This symptom cannot, therefore, be due entirely to disintegration of the membrane, for it was present in No. 2 before there was a membrane. The extreme rapidity of the pulse was also marked before the disease was clinically fully developed, and it clearly betokened something more than an ordinary sore throat. As regards the after effects of the serum, it will be seen that they were well marked in No. 1 case, which had three injections, or in all, 30 cc. of anti-toxic serum. More transient in case No. 2, which had two injections, or 22 cc. of serum. They were absent altogether in the third case. It will be interesting to observe if it is common for these symptoms to be accompanied with albuminuria, and what may be the significance thereof. Notwithstanding these drawbacks to the use of anti-toxin, it would appear in these cases at least there was (as Dr. Woodhead has already stated in others) "fall in the temperature, improvement of the pulse, disappearance of the membrane, complete recovery. No sequelæ." These good results I attribute to the curative serum, and the unremitting care of the nurses in seeing that the patients took all the nourishment ordered, &c. It is, I think, by publishing such cases as these that the true merits of the serum will be elicited. Everything depends upon the time when cases first come under treatment. The earlier the better, so that the diphtheritic poison is neutralized before degeneration of the tissues has taken place.

